# Nursing Progress and Its Developments: III. Nursing as a Science

**Published:** 1917-12-22

**Authors:** 


### THE MATRONS' AND SISTERS' DEPARTMENT.

### NURSING PROGRESS AND ITS DEVELOPMENTS.

### III. Nursing as a Science.

Every science is built up of a multiplicity of observed facts. Every science in the course of development has to go through an empirical period during which the relation of these facts to one another, and their bearing on the truths it is sought to elucidate, are imperfectly understood. At present nursing as a science is still in the making. Matrons and sisters in every training-school worthy of the name are experimenting and observing, with the object in view of resolving all the countless details which make up the art of modern nursing into a homogeneous whole. It used to be, and no doubt still is, matter of common complaint by matrons and the heads of nursing institutes that women were often to be encountered holding the certificates of famous hospitals, who had succeeded in slipping through the mesh without performing or even seeing performed some of the commonest nursing processes.

While that is the case, so long as that is possible, nursing cannot be considered an organised profession. One of the main services for which nurses look to the College of Nursing is such a reorganisation of system that all probationers may be assured of competent instruction in all the essential details comprised in their calling. It is probably no longer possible for a probationer to be kept for eighteen months of her course cutting bread-and-butter or counting out linen. But it remains true to this day that the precautions which are taken in most training-schools to ensure that probationers receive sound teaching and opportunity for practice in every ordinary branch of nursing are insufficient. If they do not depend, as they do in many institutions, on the matron's or sister's memory, or on the determination of the probationer herself to leave no gaps in her training, they are frequently such as to leave the probationer at the mercy of any sudden occurrence, such as the closing of certain wards in the hospital, a change of matron, a period of sick leave, or the like.

The first step to be taken in ensuring equality of opportunity up to a certain stage for each probationer is to classify and arrange the enormous mass of practical details which have to be mastered. We have already had occasion to observe that the Nightingale School has now in use a system which carries the probationer a long way towards the required goal. The matrons who are actively promoting the College of Nursing are precisely those who not only theoretically, but in the working of their own wards, are striving to this end. They know from experience the difficulties involved in adjusting any central system, to the requirements of institutions differing widely one from another, and thus they may be safely trusted, as no matrons resting on the experience of many years gone by can possibly be trusted, to frame such an organic whole out of the piecemeal fragments of nursing lore now imparted as shall not prove either unworkable or disintegrating as a system to the hospitals which adopt it.

More than this. These matrons working for the good of the nursing profession are women strongly imbued with the essence of British nursing ideals, humanity towards the patients. It is by no means certain that in every country the precision and technical alertness attained by a central system have been achieved without some loss of this inner fragrance. Theorists sitting round a table may easily consider it outside their sphere, and by ignoring it may do fatal mischief. But the matrons who are striving to found the College of Nursing on a basis of professional efficiency are conspicuous in their own institutions for that priceless quality which draws the heart of the wounded, and indeed of all who suffer, to those who bear the uniform. Among all the healing influences which science brings in its train it may be we shall come to recognise in days to come that humanity takes the front place.

